# Two Cases of Granular Degeneration of the Cervix Uteri

**Published:** 1886-11

**Authors:** 


### *TWO CASES OF GRANULAR DEGENERATION OF THE CERVIX UTERI TREATED WITH S. H. KENNEDY'S EXTRACT OF PINUS CANADENSIS.*

Dr. Gray, F. R. C. P., Edinburg, says: A short time ago I procured from the Rio Chemical Company some of the above drug, and also of the Aletris Cordial. I had at the time two patients under treatment for granular degeneration of the cervix uteri, also known as granular ulcer. The first case, a multipara, aged thirty, had given birth to her first child eight years ago, an interval of seven years without any pregnancy elapsed, owing to leucorrhœa and chronic endometritis, which were cured by the ordinary treatment, occupying however, nearly twelve months. After being cured, pregnancy of her second child occurred; but again, within six weeks after the birth of this child, in November, 1885, the granular degeneration manifested itself, and cervical catarrh, for which I put her upon the usual treatment, iodised phenol, glycerine tampons, douche of water by the syphon uterine douche, etc., but slow progress was made. Still, progress was made. On receipt of the Ext. Pinus, I directed my patient to mix a tablespoonful of it with water and use as a douche for two days. On my next visit, I twisted a bit of cottonwool around a Playfair probe, dipped it in the pure Ext. Pinus, and, after cleansing the mucus from the cervix, applied the probe, charged with the extract to the canal; I then made a tampon and saturated it with the extract and applied it direct to the cervix; in two days removed and applied a fresh tampon saturated as before; then again passed Playfair's probe, armed as before; afterwards directed my patient to use the extract with the douche. Within fourteen days from the first application every trace of cervical catarrh and erosion had disappeared.

*Second Case.*—H. G., aged forty, multipara, gave birth to her seventh child in September, 1885; no uterine trouble until December, when I examined her for pains and leucorrhœa; found degeneration of cervix with slight endometritis and hyperæmia of cervix; applied tampons saturated with glycerine; gave her ergot and cinchona, etc.; used the douche for some time, but without any pronounced benefit. I applied S. H. Kennedy's Extract of Pinus Canadensis, as in former case. In addition, I gave her the Aletris Cordial on account of the thickened hyperæmic state of the cervix, and, in the course of ten days, she was completely cured by these remedies. I think in Pinus Canadensis we have a good astringent, with special healing properties for the uterus. The Aletris Cordial certainly seems to act as a uterine alterative and tonic.—*Medical Press.*

